# Reactive astrogliosis in the era of single-cell transcriptomics

**DOI:** 10.3389/fncel.2023.1173200

**Published:** 2023-04-20

**Authors:** Zuzana Matusova, Elly M. Hol, Milos Pekny, Mikael Kubista, Lukas Valihrach

**Affiliations:** ^1^Laboratory of Gene Expression, Institute of Biotechnology of the Czech Academy of Sciences, Vestec, Czechia; ^2^Faculty of Science, Charles University, Prague, Czechia; ^3^Department of Translational Neuroscience, University Medical Centre Utrecht Brain Centre, Utrecht University, Utrecht, Netherlands; ^4^Laboratory of Astrocyte Biology and CNS Regeneration, Center for Brain Repair, Department of Clinical Neuroscience, Institute of Neuroscience and Physiology, Sahlgrenska Academy at the University of Gothenburg, Gothenburg, Sweden; ^5^Florey Institute of Neuroscience and Mental Health, Parkville, VIC, Australia; ^6^University of Newcastle, Newcastle, NSW, Australia; ^7^TATAA Biocenter AB, Gothenburg, Sweden; ^8^Department of Cellular Neurophysiology, Institute of Experimental Medicine of the Czech Academy of Sciences, Prague, Czechia

**Keywords:** reactive astrogliosis, astrocytes, cell populations, single-cell RNA-seq, neuroinflammation, neurodegeneration, CNS diseases

## Abstract

Reactive astrogliosis is a reaction of astrocytes to disturbed homeostasis in the central nervous system (CNS), accompanied by changes in astrocyte numbers, morphology, and function. Reactive astrocytes are important in the onset and progression of many neuropathologies, such as neurotrauma, stroke, and neurodegenerative diseases. Single-cell transcriptomics has revealed remarkable heterogeneity of reactive astrocytes, indicating their multifaceted functions in a whole spectrum of neuropathologies, with important temporal and spatial resolution, both in the brain and in the spinal cord. Interestingly, transcriptomic signatures of reactive astrocytes partially overlap between neurological diseases, suggesting shared and unique gene expression patterns in response to individual neuropathologies. In the era of single-cell transcriptomics, the number of new datasets steeply increases, and they often benefit from comparisons and integration with previously published work. Here, we provide an overview of reactive astrocyte populations defined by single-cell or single-nucleus transcriptomics across multiple neuropathologies, attempting to facilitate the search for relevant reference points and to improve the interpretability of new datasets containing cells with signatures of reactive astrocytes.

## 1. Introduction

Astrocytes carry out functions essential for maintaining homeostasis in the CNS. They induce and control neuronal synapses, supply neurons with energy, and participate in neural plasticity mechanisms. Astrocytes also support the blood–brain barrier integrity and function and can react to cytokines circulating in the vascular system (Pekny and Pekna, 2014; Hasel and Liddelow, 2021; Lee et al., 2022). In disease or upon injury, astrocytes respond by reactive astrogliosis. This is characterized by increased expression of immune response genes and cytoskeletal and extracellular matrix (ECM) components, which is followed by morphological and functional changes (Wilhelmsson et al., 2006; Pekny et al., 2016; Escartin et al., 2021).

Reactive astrocytes are classically identified by hypertrophy of cellular processes and increased expression of the glial fibrillary acidic protein (GFAP) (Wilhelmsson et al., 2006; Hol and Pekny, 2015; Escartin et al., 2021). Although astrocytes show some common features in response to different pathological stimuli, their heterogeneity is far more complex and dynamic than previously thought (Stahlberg et al., 2011; Rusnakova et al., 2013; Orre et al., 2014; Pekny et al., 2019; Batiuk et al., 2020; Bayraktar et al., 2020; Gomes et al., 2020; Habib et al., 2020; Wheeler et al., 2020; Hasel et al., 2021; Sadick et al., 2022). We and others have recently cautioned against using a single or only a few selected marker genes to define astrocyte reactive states (Escartin et al., 2021). Instead, reactive populations of astrocytes should be defined considering multiple parameters, including gene expression, proteomics, morphology, and function. In addition, the astrocyte phenotype is determined by location in the CNS, disease pathogenesis and progression, co-morbidities, age, sex, and other sources of heterogeneity.

High-throughput single-cell and single-nucleus RNA sequencing (scRNA-seq/snRNA-seq), along with spatial transcriptomics, have been widely used to determine the diverse cellular composition of the CNS and to classify subsets of cells. These innovative technologies also allow for the determination of the diverse populations of cell types and cell states involved in the pathogenesis of brain disorders (Keren-Shaul et al., 2017; Habib et al., 2020; Kenigsbuch et al., 2022). The analysis of transcriptomic data often employs reference datasets, when annotating cellular clusters and uses data integration to increase resolution and identify concordances with previous studies (Stuart et al., 2019). The existing transcriptomic databases gather data obtained through various protocols from a wide range of specimens, allowing for the exploration of pre-processed data [e.g., PanglaoDB by Franzen et al. (2019)] or browsing through an easily accessible collection of references along with key metadata [e.g., single-cell studies database by Svensson et al. (2020)]. However, as the number of available datasets grows, it becomes important, albeit difficult, to search for relevant reference points.

**Figure 1:**
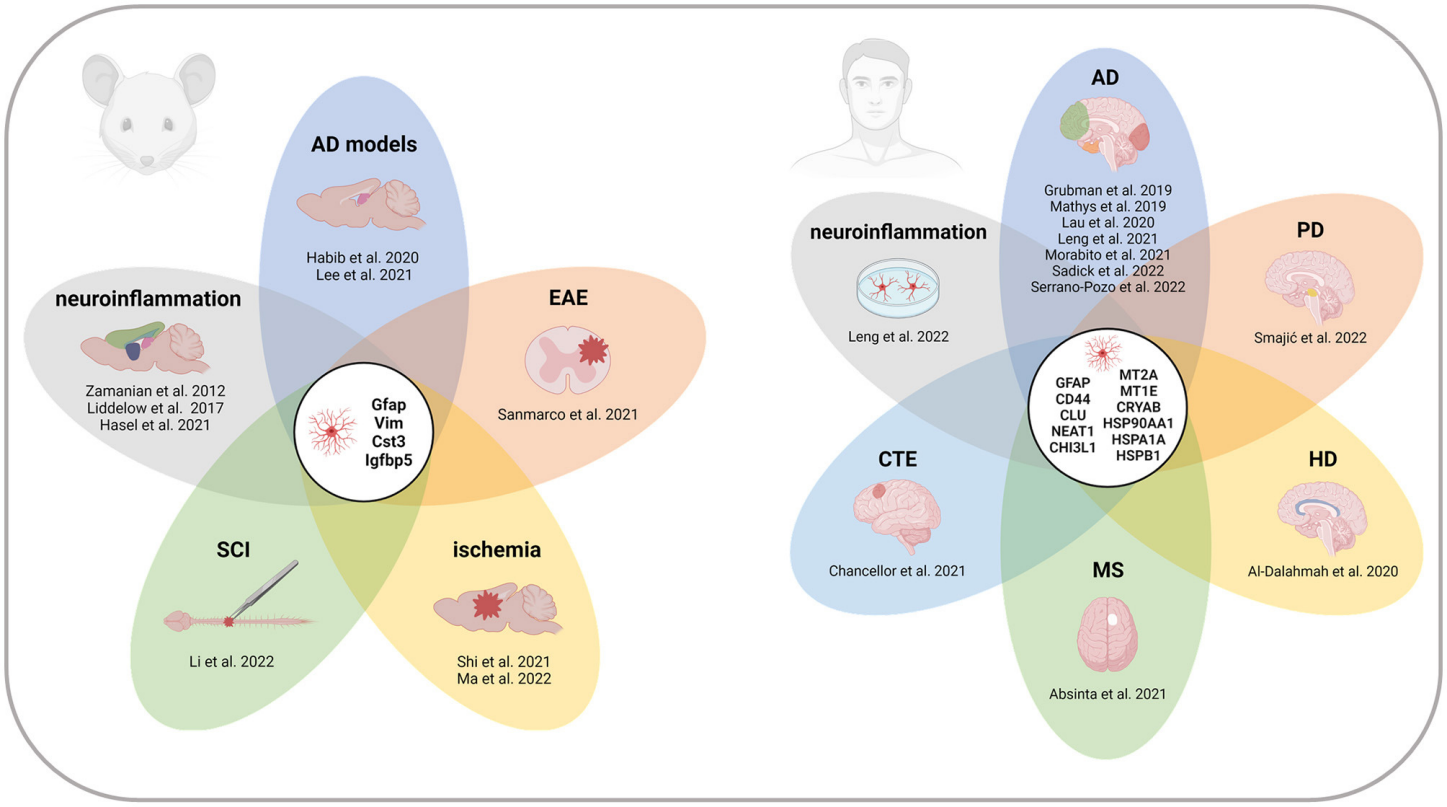
Recurrent marker genes of reactive astrocytes across CNS regions and pathologies in mice and humans. Genes indicated in the centers of the diagrams appeared in at least four of the studies listed in Table 1. All the references report at least one of the recurrent genes. References without overlap are not included in the diagrams. Note that the gene signatures of individual reactive populations are not exhaustive, and therefore, the presented gene overlap cannot be generalized as a complete list of reactive astrocyte markers. AD, Alzheimer's disease; CTE, chronic traumatic encephalopathy; EAE, experimental autoimmune encephalomyelitis; HD, Huntington's disease; MS, multiple sclerosis; PD, Parkinson's disease; SCI, spinal cord injury. Created with BioRender.com.

Here, we present an overview of reactive astrocyte populations described in neurodegenerative diseases, acute injuries, and other pathological conditions using scRNA-seq and snRNA-seq technologies in rodents and humans. We summarize the key studies and the marker genes defining the populations of reactive astrocytes (Table 1, Supplementary Table 1) and identify the core of reactive astrocyte-associated genes that are repeatedly listed across datasets (Figure 1). We believe this compact resource will help to identify relevant reference datasets to study reactive astrocytes, and thereby contribute to a better understanding of astrocyte heterogeneity and function in disease.

**Table 1:** Gene signatures of selected populations of reactive astrocytes (mostly based on single-cell studies).

**Study**	**Cluster**	**Gene signature**	**Notes**
Zamanian et al. (2012) and Liddelow et al. (2017)	Pan-reactive	*Lcn2, Steap4, S1pr3, Timp1, Hsbp1, Cxcl10, Cd44, Osmr, Cp, Serpina3n, Aspg, Vim, Gfap*	Reactive signatures as in Liddelow et al., 2017, Figure 1a
	A1	*H2.T23, Serping1, H2.D1, Ggta1, Iigp1, Gbp2, Fbln5, Ugt1a, Fkbp5, Psmb8, Srgn, Amigo2*	
	A2	*Clcf1, Tgm1, Ptx3, S100a10, Sphk1, Cd109, Ptgs2, Emp1, Slc10a6, Tm4sf1, B3gnt5, Cd14*	
Leng et al. (2022)	IRAS1	*FTH1, MT2A, CXCL1, CCL2, CXCL8, CCL20, CXCL6, CXCL3, C15orf48, MT1E, LGALS1, CSF3, SOD2, CXCL2, PTX3, SERPINE1, TMSB10, SAA1, IL32, SRGN*	Top 20 genes from Supplementary Table 4a, cluster 1, ordered based on avg_diff
	IRAS2	*FTH1, TMSB10, CCL2, IL32, B2M, TMSB4X, LGALS1, SOD2, MT-ND3, HLA-B, NNMT, S100A11, PFN1, PRELID1, CALR, RPS27A, PSME2, CYP1B1, ICAM1, NFKBIA*	Top 20 genes from Supplementary Table 4a, cluster 2, ordered based on avg_diff
Hasel et al. (2021)	Cluster 4	*Lcn2, Ifitm3, Timp1, B2m, Vim, Gfap, Psmb8, Bst2*	Top genes enriched in cluster as in Figure 2c
	Cluster 8	*Igtp, Gm4951, Ifit3, Iigp1, Irgm1, Tap1, Gbp2, Ifit1, Isg15, Cxcl10*	
Habib et al. (2020)	Gfap-high	*Myoc, Gfap, Id3, Igfbp5, Aqp4, Serpinf1, Id1, Apoe, 6330403K07Rik, Slc38a1, Clu, Fbxo2, Vim, Ckb, Fabp7, Cd9, Ptchd2, Fxyd6, Unc5c, Scd2*	Top 20 genes from Supplementary Table 2, cluster 1 vs. 6, ordered by log_2_FC
	DAA	*Rpl36-ps3, Gfap, Vim, Clu, Igfbp5, Id3, Gsn, Cd9, Fxyd1, Ctsb, Apoe, C4b, Kcnip4, Plce1, Cst3, Ggta1, Serpina3n, S100a6, Cnn3, Mt2*	Top 20 genes from Supplementary Table 2, cluster 1 vs. 4, ordered by log_2_FC
Morabito et al. (2021)	*GFAP*^high^/*CHI*3*L*^+^	*CHI3L1, TPST1, RGS6, AC083837.2, ARHGEF3, ZFYVE28, ELL2, SAMD4A, GNA14, SLC44A3-AS1, IFI16, CPNE8, CD44, WWTR1, AC010655.4, ADAM12, ETV6, CLIC4, OSMR-AS1, PKNOX2*	Top 20 genes from Supplementary Data 1d, cluster ASC3, ordered by log_2_FC
Zhou et al. (2020)	Astro0, Astro1	*PDE4DIP, PFKP, COL5A3, NCAN, SLC5A3*	Figure 5c, genes upregulated in AD clusters Astro0 and Astro1
Lee et al. (2021)	A-C5a	*Serpina3n, Aqp4, Igfbp5, A2m, Gsn, Gfap, Cd44, Ctsb, Slc16a1, Ddr1, Serpinf1, C4b, Ctsd, Plat, Serping1, Timp2, Tagln3, Vim, Ccdc3, Mlc1*	Supplementary Table 8, Cohort II astrocyte
	A-C5b	*Cst3, Scrg1, Cd81, Reep5, Lxn, Tmco1, Cavin3, Nde1, Gstm5, Clu, Nudt10, Tmem50a, Gm44974, Tmem208, Cacng6, Gm12892, Gm38379, Hsd11b1, Ccdc13, Ccdc116*	Supplementary Table 8, Cohort II astrocyte
Grubman et al. (2019)	a1	*LINGO1, HSPA1A, BCYRN1, BOK, DNAJB2, MT-CO2, MT-ND3, HSP90AA1, GFAP, MT-ND1, MT-ND2, CRYAB, HSPB1, MT-CYB, MT-ATP6, MT-ND4, MT-CO3, CHI3L1, DNAJB1, AEBP1*	Top 20 genes ordered by log_2_FC as at web interface: http://adsn.ddnetbio.com/, log_2_FC > 0.5, FDR < 0.05
	a2	*XIST, MAOB, NEAT1, GOLGB1, GFAP, CD44, PLEKHA5, CH17-189H20.1, ID3, VCAN, PLCE1, HIF3A, DCLK1, RP11-507B12.2, MAP1B, GADD45G, TSHZ2, ADAMTSL3, GALNT15, RHPN1*	
Mathys et al. (2019)	Ast1	*SLC26A3, MT1G, RASGEF1B, LINGO1, MT1M, MT1E, CSRP1, KCNJ10, VIM, C10orf54, MT2A, GJA1, GLUL, TMBIM6, CLU, WFS1, PBXIP1, PPP1R1B, SDC4, HSP90AB1*	Top 20 genes from Supplementary Table 6, ordered by log_2_FC
Lau et al. (2020)	AD-up-regulated	*PTOV1, KIAA0930, PRRX1, PLCG2, ACAP3, IRS2, RIMS1, GFAP, HSPA1B, PTGES3, TTTY14, LINC00609, VCAN, DLGAP1, LINGO1, MT-ND5, DNAJB1, HSPB1, DNAJB2, HMGB1, HSPH1, CRYAB, AEBP1, FP671120.1, NEAT1, MTRNR2L12, DHFR, HSPA1A, RASGEF1B, HSP90AA1, SLC26A3, FP236383.1*	Supplementary Figure 5d, top genes enriched in a1 and a6 populations
Leng et al. (2021)	GFAP_high_	*GFAP, CD44, TNC, HSPB1, HSP90AA1*	Figures 5c, d – expression of reactivity genes; markers of the cluster are not available
Sadick et al. (2022)	Pan-astrocytic DEGs	*NEAT1, RANBP3L, PLCG2, SLC39A11, PFKP, SESN1, PLPP1, LRMDA, ABCA1, SLC25A18, RASSF8, LINC00511, WWOX, NPAS2, CPNE8*	Supplementary Table 6, overlap of DEGs in each cluster
Serrano-Pozo et al. (2022)	astR1, astR2	*ACTB, APP, AQP4, CD44, CLU, CRYAB, ECE1, FGFR1, GFAP, HSPB1, HSPB8, LAMA1, MAOB, MAP1B, MAPT, MT1E, MT2A, PRDX6, S100B, VCAN*	Commonly selected markers of reactive clusters from Figure 5b
Smajic et al. (2022)	CD44^high^	*KCNE4, SHISA9, CP, EMP1, NEAT1, CHI3L1, TPD52L1, CACNB2, HPSE2, ATRNL1, HS6ST3, MYRIP, PLCXD3, SLCO3A1, ST6GAL1, MAN1C1, NFASC, CERS6, SYNPO2, AEBP1, DCLK1, CD44, GALNT15, S100A6, CRYAB, FTH1, CPAMD8, C8orf34, TTN, NRXN3, HSP90AA1, HSPA1A, HSPB1, UBC*	Figure 4F, astrocytic DEGs upregulated in PD overlapped with genes enriched in the astrogliosis trajectory toward CD44^high^
Al-Dalahmah et al. (2020)	Cluster 1	*MT2A, MT1F, MT1E, MT1X, MT1G, MT1M, FTL, MT3, FAM171B, TPD52L1, EGLN3, CPE, GJB6, EPHX1, TKT, CLU, RGCC, NUPR1*	Table 2, selected markers
	Cluster 2	*HSP90AA1, HSPB1, CRYAB, MTATP6P1, HSPA1A, UBC, CALM1, HSP90AB1, ATP1B1, GPM6B, CCK, VAMP2, MAP1A, NRGN, EEF1A1, EIF1, TMSB4X*	
	Cluster 5	*MALAT1, GFAP, NEAT1, FP236383.2, SORBS1, PLP1, CEBPD, TSC22D1, PRRG3, SP100, HILPDA, COL6A3, RASD1, CHI3L1, SLC14A1, SAT1, HNRNPH1, PCDH9, PRKCA, BCL6, LINC00969, ANKRD36B, HIF3A, ITGB8, DTNA, HSPA1B, ETNPPL*	
	Cluster 6	*ID2, S100B, FOS, DBI, HES1, JUNB, FABP7, H3F3B, ID3, PTGDS, MT1H, IFITM3*	
Wheeler et al. (2020)	MAFG	*Fyb, C1qc, Ccl4, Ly86, Lpcat2, Tyrobp, C1qa, Cyba, Rgs10, Ctsc, Cx3cr1, Bcl2a1b, Ccl2, Selplg, Hexb, Fcer1g, Cebpb, Ctsh, Mpeg1, Gm8995*	Top 20 genes from Supplementary Table 3, cluster 5, ordered by log_2_FC, p_adj_ < 0.05
Sanmarco et al. (2021)	LAMP1^+^TRAIL^+^	*Fscn1, Egr1, Ier2, Tnfaip3, Il1a, Nfkbia, Btg2, Marcksl1, Gadd45b, Jun, Cst3, Cd83, Fos, Nfkbiz, Icam1, Ier5, Junb, Ppp1r15a, Tob2, Sparc*	Top 20 genes from Supplementary Table 8, cluster 0, ordered by log_2_fc, p_adj_ < 0.05
Absinta et al. (2021)	AIMS	*IGKC, VIM, APOE, CLU, CST3, ACTG1, FTL, CAPS, ACTB, CD81, ITM2C, SRP14, CEBPD, S100B, ZFP36L2, AGT, TUBA1A, SPARC, GAPDH, CKB*	Top 20 genes from Supplementary Table 7, cluster 6, ordered by log_2_FC
	Reactive/stressed astrocytes	*NAMPT, PLOD2, HSPH1, HILPDA, TPST1, ELL2, TRIO, ARID5B, ABCA1, ZFYVE28, SERPINH1, GPR158, HSP90AA1, SAMD4A, CNN3, ACTN1, ARHGEF3, NOD1, FAM189A2, SPOCK1*	Top 20 genes from Supplementary Table 7, cluster 1, ordered by log_2_FC
		*RASGEF1B, MT-ND2, SLC26A3, MT-CO3, MT-ND1, MT-ND3, MT-CO2, MT-CO1, MT-ND4, MT-CYB, MT-ATP6, AC105402.3, MT-ND5, NEAT1, BNIP3L, LINC00511, SLC16A1-AS1, GNB1, GOLGA4, SGMS1*	Top 20 genes from Supplementary Table 7, cluster 5, ordered by log_2_FC
Shi et al. (2021)	Cluster 3	*Apod, Ttr, Ier2, Igfbp5, Btg2, Cpe, Spp1, Txnip, Dnajb1, Cyr61, Klf2, Egr1, Gstm1, Mt1, Mt2, Nfkbia, Zfp36, Sox9, Junb, Cst3*	Top 20 genes from source data for Figure 10f, ordered by log_2_FC, p_adj_ < 0.05
Ma et al. (2022)	AST12_C	*Plp1, Ptgds, Gfap, Mal, Mbp, Cldn11, Apod, Hsbp1, S100a10, Mobp*	Top 10 markers in Figure 3i
AST24_C	*Spp1, Ccl14, Gfap, Cd14, Ccl12, Ccl3, Cxcl12, Lgals3, C5ar1, Ccl6*	Top 10 markers in Figure 4i
Zamboni et al. (2020)	AC3	*Stat1, Bst2, Iigp1, Ifitm3, H2-T23, H2-D1*	Figure 3b, selected markers
Li et al. (2022)	Cluster 4	*Aqp4, Gfap, S100a6, Vim, Nes*	Cluster signature genes from Figure 4b
Cluster 5	*S100a6, Vim, Nes, Tubb5, Mki67*	
Wang et al. (2021)	Cluster 4	*Spp1, Cd44, Srbd1, Smarcd2, Slc16a3, Acsl4, Ccl12, Lrp1, Pvr*	Supplementary Data Sheet 1, ordered by log_2_FC, p_adj_ < 0.05
Chancellor et al. (2021)	Astrocyte2	*SLC14A1, HIF3A, PDK4, LINC01088, NPL, ZHX3, ADGRV1, PPP2R2B, KANSL1, CDH20, PARD3, MAPK10, MAGI2, PPM1E, CYP4F3, GRAMD2B, ECHDC2, MIR99AHG, RORA, ARL17B*	Top 20 genes from Supplementary File 3, Table 8, ordered by avg_diff
		Astrocyte3	*SERPINA3, CD44, BCL6, GLIS3, IRS2, CHI3L1, CADPS, ANGPTL4, CLU, MAPK4, GLUL, VEGFA, ARHGEF3, CSNK2A1, MAN1C1, MT2A, EFCAB3, SIAH1, PFKP, KCTD21-AS1*	

AIMS, astrocytes inflamed in multiple sclerosis; avg_diff, average difference; DAA, disease-associated astrocytes; DEGs, differentially expressed genes; FDR, false discovery rate; log_2_FC, log_2_ fold change; p_adj_, adjusted p-value; PD, Parkinson's disease. The signatures have been presented in the original studies, and the source is indicated in the notes. If additional filtering was applied to prioritize the genes, the thresholds are noted.

## 2. Neuroinflammation

Zamanian et al. (2012) provided a landmark transcriptomic study defining distinct states of reactive astrocytes emerging during neuroinflammation and ischemic stroke in mice injected with bacterial lipopolysaccharide (LPS) and in the middle cerebral artery occlusion (MCAO) model. Reactive astrocytes in both conditions upregulated intermediate filament proteins (known also as nanofilament proteins), ECM, and adhesion molecules, regulators of metal ion homeostasis, and components of immune response pathways. However, while LPS reactive astrocytes showed signs of an elevated immune response, MCAO astrocytes upregulated genes involved in metabolic activity and proliferation, presumably connected with post-stroke neuroplasticity responses.

Lipopolysaccharide and MCAO reactive astrocytes, occasionally still termed A1 and A2 (Liddelow et al., 2017; Escartin et al., 2021), have become perceived as detrimental/pro-inflammatory or beneficial/anti-inflammatory subtypes. A1-like astrocytes were found to be induced by the cytokine trio Il-1α, TNF, and C1q secreted by microglia and were identified in *post-mortem* samples from patients suffering from Alzheimer's disease (AD), Parkinson's disease (PD), amyotrophic lateral sclerosis (ALS), and multiple sclerosis (MS) (Liddelow et al., 2017; Guttenplan et al., 2020). Although such a binary division is now accepted as oversimplified and insufficient to encompass the real spectrum of reactive astrocyte heterogeneity (Escartin et al., 2021), these early experiments provided a useful point of reference for later studies.

Using single-cell transcriptomics, Leng et al. (2022) explored the heterogeneity of astrocytes derived from human-induced pluripotent stem cells (hiPSCs) treated by the cytokine trio used by Liddelow et al. (2017) and defined two clusters of neuroinflammation-induced reactive astrocytes. While the IL-1/IL-6-responsive astrocytes (IRAS1) were characterized by acute phase response genes, the TNF/IFN-responsive astrocytes (IRAS2) upregulated interferon signaling, indicating their specialized function. Using an integrative analysis, both signatures were identified in an MS mouse model (Wheeler et al., 2020), in hiPSC-derived astrocytes stimulated with cytokines (Barbar et al., 2020), and in a study by Hasel et al. (2021).

Hasel et al. (2021) addressed the astrocyte response in LPS-induced neuroinflammation in mice. A neuroinflammation-related fast-responding cluster was found almost exclusively in the LPS samples. As IRAS2, it was induced by IL1α, TNF, and C1q cytokines, regulated by Stat1 and Stat2, and showed differential expression of genes involved in the interferon response, antigen processing, and antigen presentation genes. Noteworthy, these astrocytes were localized close to blood vessels and around ventricles, suggesting their region-specific function, which cannot be observed in cell cultures (Leng et al., 2022). The signature of this cluster was found to be expressed also in models of AD (Habib et al., 2020; Zhou et al., 2020), MS (Wheeler et al., 2020), and stab wound injury (Zamboni et al., 2020). Another interesting cluster localized in the white matter (WM), was present in control and treated mice, and upon LPS treatment activated the expression of inflammatory genes. Together, these studies indicated that, in response to neuroinflammation, astrocytes form functionally and regionally diverse reactive populations.

## 3. Alzheimer's disease

The experimental neuroinflammation model provided valuable insights into astrocyte heterogeneity and predicted potentially higher complexity in neurodegenerative diseases. This has been confirmed by extensive AD research, as discussed in the following paragraphs. For a comprehensive overview of sc-/snRNA-seq AD studies without focus on astrocytes, refer to Cuevas-Diaz Duran et al. (2022) and Saura et al. (2022), or explore the scREAD database (Jiang et al., 2020).

In a key study defining astrocyte reactivity in AD, Habib et al. (2020) analyzed the hippocampus of the 5xFAD mouse using snRNA-seq. The authors identified two clusters of astrocytes with increased expression of *Gfap*, one of which was more abundant in 5xFAD samples. Accordingly, these were termed disease-associated astrocytes (DAAs). The DAAs partially shared the expression profile with Gfap-high astrocytes that were also present in the control samples, but the DAAs specifically upregulated genes related to endocytosis, complement, aging, and amyloid metabolism. Furthermore, the presence of homeostatic and intermediate populations suggested dynamic transitions of individual astrocyte states. DAA-like populations were also found in 5xFAD cortices and aged brains of mice and humans (Mathys et al., 2019), indicating that the signature is not specific to a single AD mouse model or brain region. These results were extended by Morabito et al. (2021), who detected Gfap-high and DAA signatures enriched among nuclei from prefrontal cortices of AD patients and using chromatin accessibility data, they identified *FOSL2* as a potential regulator of the DAAs. Moreover, they found a corresponding cluster of *GFAP*^high^/*CHI*3*L*^+^ astrocytes proportionally increased in AD.

Zhou et al. (2020) noticed inter-species differences in astrocyte changes between the 5xFAD mouse model and the prefrontal cortex of AD patients. While only a limited transcriptional change was reported in mice, several astrocyte clusters differed in proportions between human controls and AD samples. Of note, different genotypes of the microglial receptor TREM2, a known AD risk factor (Wolfe et al., 2018), were found to not affect reactive astrogliosis. Another analysis of the hippocampus of the AD mouse models of amyloidosis (PS2APP) and amyloidosis combined with tauopathy (TauPS2APP) revealed the presence of reactive astrocytes (Lee et al., 2021). Their comparison across conditions showed comparable transcriptional changes not only in PS2APP and TauPS2APP but also in a *Trem2* knockout, indicating that astrocytes respond similarly in different models of AD and in a TREM2-independent fashion.

Focusing on AD patient samples, three early studies investigated the entorhinal cortex—the site of early pathological changes (Grubman et al., 2019) and the prefrontal cortex (Mathys et al., 2019; Lau et al., 2020) affected later in the disease progression. Interestingly, populations of AD-associated astrocytes identified in individual studies showed overlapping gene signatures, illustrated by common upregulation of heat shock proteins, *LINGO1* or *RASGEF1B*. These results highlighted conserved transcriptomic changes in different brain regions affected in the early and late AD pathogenesis. This has been confirmed by an independent study comparing the entorhinal cortex and the superior frontal gyrus within the prefrontal cortex of AD patients at different stages of the pathology, identifying GFAP_high_ astrocytes in comparable abundance across regions and stages (Leng et al., 2021).

Using an improved enrichment strategy (based on LHX2^+^NeuN^−^ sorting), Sadick et al. (2022) increased the astrocyte proportion from ≈ 15% of nuclei in previous studies (estimated by the authors) to over 50%, allowing for an in-depth analysis of AD prefrontal cortices. Interestingly, no cluster was identified as disease-related; instead, each showed individual changes in AD samples. The global transcriptomic alteration in all astrocytes was represented by pan-astrocytic differentially expressed genes. Furthermore, multiple clusters upregulated metallothioneins, heat shock proteins, and other previously reported reactivity-related genes. Thus, reactive astrocytes might not be limited to a single population, but rather originate in multiple homeostatic populations.

A similar conclusion was reached in a comprehensive study by Serrano-Pozo et al. (2022), who analyzed five brain regions consecutively affected during AD progression. The authors identified two reactive astrocyte clusters characterized by a reactive signature. Importantly, analyses of multiple intermediate clusters and trajectories supported the homeostatic origin of reactive astrocytes, as proposed earlier by Habib et al. (2020) and Sadick et al. (2022).

## 4. Other proteinopathies

Alzheimer's disease, PD, and Huntington's disease (HD) belong to neurodegenerative proteinopathies. PD and HD have also been analyzed with single-cell transcriptomics (Ma and Lim, 2021; Malla et al., 2021), but only a few studies have focused on astrocytes. Other proteinopathies with an important or causative role of astrocytes remain to be investigated, e.g., Alexander disease (Pajares et al., 2023).

In the midbrain of PD patients, Smajic et al. (2022) identified CD44^high^ astrocytes, representing the terminal activated state in astrogliosis trajectory. Astrocytes originating in PD patients were enriched at the end of this activation trajectory, suggesting their role in PD-associated neuroinflammation. Al-Dalahmah et al. (2020) analyzed the cingulate cortex of patients suffering from HD and identified three HD clusters of astrocytes, expressing variable levels of metallothioneins and *GFAP*, possibly transitioning one into another. Interestingly, similarly to Habib et al. (2020), they also identified *GFAP* high astrocytes in controls, implying the existence of astrocyte heterogeneity even in healthy individuals. Collectively, these PD and HD studies together with the AD studies show the presence of similar reactive astrocyte populations across multiple disorders.

## 5. Multiple sclerosis

Although single-cell transcriptomic studies of MS have focused mainly on neurons, microglia, and oligodendrocytes (Falcao et al., 2018; Jakel et al., 2019; Schirmer et al., 2019), astrocyte heterogeneity has not remained unnoticed [reviewed in Lo et al. (2021)]. Using a model of experimental autoimmune encephalomyelitis (EAE), Wheeler et al. (2020) identified a population of astrocytes expressing pro-inflammatory genes. A similar population was also found in active WM lesions in MS patients. Due to high levels of the transcriptional regulator MAFG, this population was termed MAFG-driven astrocytes. Integration revealed the presence of MAFG astrocytes in other MS datasets, regardless of the CNS region analyzed (Lake et al., 2018; Jakel et al., 2019; Schirmer et al., 2019). Soon after, Hasel et al. (2021) and Leng et al. (2022) detected the signature of the neuroinflammatory populations of astrocytes in the dataset produced by Wheeler et al. (2020), showing a shared response of mouse and human astrocytes in experimental neuroinflammation and MS.

A follow-up study identified the LAMP1^+^TRAIL^+^ astrocyte population characterized by anti-inflammatory and pro-apoptotic properties, which was depleted in EAE samples (Sanmarco et al., 2021), and was proposed to act protectively. The impaired balance of MAFG and LAMP1^+^TRAIL^+^ astrocytes in EAE/MS may lead to outnumbering of the protective astrocytes by the pro-inflammatory population, likely exaggerating the inflammatory environment in the affected tissue.

Considering the spatial aspect of MS, Absinta et al. (2021) identified reactive astrocytes enriched in multiple areas around and within MS lesions, expressing heat shock proteins, cell death regulators, and mitochondrial genes. Another population of astrocytes inflamed in MS upregulated *GFAP, APOE, VIM*, and several A1 markers. It was found almost exclusively at the edges of chronic active lesions, showing that distinct reactive astrocytic populations arise in MS depending on lesion localization and its severity.

## 6. Neurotrauma and stroke

Neurotrauma and stroke represent another group of CNS disorders triggering substantial cellular heterogeneity that has been investigated by single-cell transcriptomics (Wilhelmsson et al., 2017; Moulson et al., 2021; Cao et al., 2022).

Shi et al. (2021) reported varying phagocytic properties of astrocytes in mouse models of hemorrhagic and ischemic strokes. Reactive astrocytes characterized by upregulated synapse pruning and lysosome-related processes were shown to be responsible for this difference. Although they were present in both conditions, their proportion decreased from 20% in ischemic to <2% in hemorrhagic mice, suggesting model-specific patterns in astrocyte reactivity. Another transcriptomic analysis of the early phases of ischemic stroke in mice identified a cluster of astrocytes enriched in MCAO samples (Ma et al., 2022). Interestingly, their transcriptional signature at 12 h and 24 h after stroke differed, transitioning from a state with upregulated oxidative phosphorylation and cell junction genes to a state defined by an increased immune and inflammatory response, implicating a dynamic nature of astrocyte reactivity in the early phases after the injury.

Zamboni et al. (2020) investigated the neurogenic potential of astrocytes in mouse somatosensory cortex subjected to stab wound injury. They identified a small cluster of reactive astrocytes expressing A1 and immune response genes. Importantly, trajectory analysis revealed the separation of the reactive and neurogenic branches, showing that they represent two independent and non-sequential states.

Two distinct clusters of reactive astrocytes were identified in a mouse model of spinal cord injury (SCI) (Li et al., 2022). Both were defined by an increased expression of *Gfap, Vim*, and *Nes*, but one cluster also expressed *Mki67* at high levels, indicating that some reactive astrocytes are proliferative, peaking 3 days after the injury. The SCI in a rat model induced a reactive astrocyte population expressing immune response-related genes (Wang et al., 2021), such as *Cd44*, which has been associated with reactive astrocytes in neurodegeneration (Grubman et al., 2019; Lee et al., 2021; Leng et al., 2021; Smajic et al., 2022), and *Spp1*, which characterizes ischemia-induced astrocytes (Shi et al., 2021; Ma et al., 2022). Collectively, these represent examples of shared reactive signatures in different pathophysiological conditions and species.

The heterogeneity of reactive astrocytes has been investigated also in chronic traumatic encephalopathy (CTE), a tauopathy induced by repetitive head impacts (Chancellor et al., 2021). The authors identified two astrocyte populations enriched in CTE. While one population was characterized by genes associated with a dysfunctional metabolism, the other expressed genes associated with neuroinflammation and reactivity. In addition to CTE, traumatic brain injury can increase the risk of neurodegenerative diseases or induce post-traumatic epilepsy, with astrocytes playing a pivotal role in these secondary processes (Graham and Sharp, 2019; Small et al., 2022). The exact mechanisms remain to be elucidated, but the identification of astrocyte genes responsible for the onset of post-traumatic complications might largely contribute to the discovery of biomarkers for severity prediction, their modulation, and mitigation of deleterious consequences.

## 7. Inter-species comparison

Several single-cell transcriptomic studies indicate shared signatures of reactive astrocytes across neurological diseases, e.g., MS, AD, and neurotrauma (Hasel et al., 2021; Leng et al., 2022). Moreover, overlapping signatures were found in human and mouse reactive astrocytes by Habib et al. (2020) and Leng et al. (2022), while others also noticed inter-species differences (Zhou et al., 2020).

To characterize shared and distinct aspects of astrocyte reactivity across species, Li et al. (2021) compared the responses of mouse and human astrocytes to various pathological stimuli. While they found partial conservation of astrocyte gene expression, they also reported significant differences in the expression of genes involved in mitochondrial metabolism and defense response. Human astrocytes were susceptible to oxidative stress damage, which in mice was mitigated by efficient detoxification and mitochondrial resilience. Furthermore, in hypoxia, mouse astrocytes promoted neuronal growth and repair, representing an advantage in regeneration capacity. Human astrocytes, on the other hand, upregulated inflammatory response and antigen presentation upon stimulation with a TNFα and double-stranded RNA. Further comparison with AD and MS suggested that the inflammatory response of astrocytes is a common denominator in multiple neuropathologies.

While mouse models provide important insight into the biology of reactive astrocytes, inter-species differences must be considered when extrapolating mouse data to human neurological diseases.

## 8. Conclusion

We provided an overview of single-cell transcriptomic studies characterizing reactive astrocytes in various CNS diseases to facilitate the interpretation of new datasets and to improve our molecular understanding of reactive astrogliosis. There is a shared “reactive signature” in multiple pathologies, suggesting the existence of a “pan-reactive” expression program, which is partly conserved across species. Integration and meta-analyses are needed to understand the similarities and differences and to assess their translational potential. Genes expressed in reactive astrocytes were also found in other glial cells, including microglia and oligodendrocytes (Keren-Shaul et al., 2017; Wilhelmsson et al., 2017; Lee et al., 2021; Kaya et al., 2022; Kenigsbuch et al., 2022), implicating common activation mechanisms also across cell types. Future studies focusing on cellular populations will characterize context-specific formation and functions of reactive astrocytes and describe their interplay with other cell types. Large-scale analyses of single-cell datasets could then identify targets for interventions, facilitate the search for suitable drug candidates (Xu et al., 2021), and improve the treatment of acute and chronic CNS diseases.

## Author contributions

ZM and LV wrote the manuscript and prepared the figures. EH, MP, and MK reviewed and edited the complete manuscript. All authors have read and agreed on the final version of the manuscript.
