# Corrigendum: Reactive astrogliosis in the era of single-cell transcriptomics

**DOI:** 10.3389/fncel.2023.1212975

**Published:** 2023-05-15

**Authors:** Zuzana Matusova, Elly M. Hol, Milos Pekny, Mikael Kubista, Lukas Valihrach

**Affiliations:** ^1^Laboratory of Gene Expression, Institute of Biotechnology of the Czech Academy of Sciences, Vestec, Czechia; ^2^Faculty of Science, Charles University, Prague, Czechia; ^3^Department of Translational Neuroscience, University Medical Centre Utrecht Brain Centre, Utrecht University, Utrecht, Netherlands; ^4^Laboratory of Astrocyte Biology and CNS Regeneration, Center for Brain Repair, Department of Clinical Neuroscience, Institute of Neuroscience and Physiology, Sahlgrenska Academy at the University of Gothenburg, Gothenburg, Sweden; ^5^Florey Institute of Neuroscience and Mental Health, Parkville, VIC, Australia; ^6^University of Newcastle, Newcastle, NSW, Australia; ^7^TATAA Biocenter AB, Gothenburg, Sweden; ^8^Department of Cellular Neurophysiology, Institute of Experimental Medicine of the Czech Academy of Sciences, Prague, Czechia

**Keywords:** reactive astrogliosis, astrocytes, cell populations, single-cell RNA-seq, neuroinflammation, neurodegeneration, CNS diseases

In the published article, there was an error in the **Funding** statement. The authors did not provide the reference to the funding provided by “la Caixa” Foundation. The correct **Funding** statement appears below.

“LV, ZM, and MK have been supported by the Ministry of Education, Youth, and Sports, under the frame of EJP RD, the European Joint Programme on Rare Diseases: CZ.1.05/1.1.00/02.0109, RVO 86652036, the European Union's Horizon 2020 research and innovation program under the EJP RD COFUND- EJP N° 825575, Czech Science Foundation GACR 23-05327S and 23-06269S, and project LX22NPO5107 (MEYS): Financed by EU—Next Generation EU. EH has been supported by grants from Alzheimer Nederland (WE.03-2017-4), NWO Gravitation BRAINSCAPES (463002004), ZonMW Memorabel (733050816), ZonMW TOP (91217035), EJP RD ALEXANDER (JTC2019/ZonMW 463002004), and la Caixa Foundation LCF/PR/HR21/52410002 (Astromad). MP has been supported by the grants from Swedish Research Council (2017-02255, 2019-00284, and 2020-01148), the ALF (724421 and 965939), W. and M. Lundgren's Foundation, The Swedish Brain Foundation (FO2018-0252 and FO2021-0082), The Swedish Stroke Foundation, Hagströmer's Foundation Millennium, T. Söderberg's Foundations, la Caixa Foundation LCF/PR/HR21/52410002 (Astromad), and P. Eriksson's, E. Jacobson's, R., and U. Amlöv's Foundations.”

The authors apologize for this error and state that this does not change the scientific conclusions of the article in any way. The original article has been updated.

