# Supplementary material for: Reactive astrogliosis in the era of single-cell transcriptomics
Source: Front Cell Neurosci. 2023 Apr 20;17:1173200. doi: 10.3389/fncel.2023.1173200 (PMC10157076; doi:10.3389/fncel.2023.1173200)
Supplement: Supplementary file 1 [file Table_1.pdf]

Supplementary Table 1. Details of the single-cell transcriptomic studies discussed in the review, ordered based on their appearance in the text.

Study	Organism	CNS Region	Disorder	Sample Details	Method	Cluster
Zamanian et al. 2012, Liddelow et al. 2017	Mouse	Ipsilateral cortex, corpus callosum, hippocampus, striatum	LPS-induced neuroinflammation, MCAO	Age P30-35	Microarray (bulk)	A1, A2
Leng et al. 2022	Human	–	IL1α, TNF, C1q-induced neuroinflammation	hiPSC derived iA	CROP-seq 10x Chromium	IRAS1, IRAS2
Hasel et al. 2021	Mouse	Cortex	LPS-induced neuroinflammation	Age P30-P35	scRNA-seq 10x Chromium	Cluster 4 and 8
Habib et al. 2020	Mouse	Hippocampus	5xFAD	Age 7 months	snRNA-seq 10x Chromium	Gfap-high, DAAs
Morabito et al. 2021	Human	Prefrontal cortex	AD	Late stage AD	snRNA-seq, snATAC-seq 10x Chromium	*GFAP* ^{high}/*CHI3L* $^{+}$ (ASC3)
Zhou et al. 2020	Human	Prefrontal cortex	AD	AD patients carrying TREM2-CV and TREM2-R62H variants	snRNA-seq 10x Chromium	Astro0, Astro1
Lee et al. 2021	Mouse	Hippocampus	PS2APP, TauPS2APP	Age 20-22 months	scRNA-seq 10x Chromium	A-C5
Grubman et al. 2019	Human	Entorhinal cortex	AD	AD patients with range of *APOE* variants (ε2, ε3, ε4)	snRNA-seq 10x Chromium	a1, a2
Mathys et al. 2019	Human	Prefrontal cortex	AD	AD patients with varying severity of the pathology	snRNA-seq 10x Chromium	Ast1
Lau et al. 2020	Human	Prefrontal cortex	AD	AD patients with range of *APOE* variants (ε2, ε3, ε4)	snRNA-seq 10x Chromium	AD-up-regulated (a1, a6)
Leng et al. 2021	Human	Entorhinal and prefrontal cortex	AD	AD patients carrying *APOE* ε3/ε3 variant	snRNA-seq 10x Chromium	$GFAP_{high}$
Sadick et al. 2022	Human	Prefrontal cortex	AD	AD patients carrying *APOE* ε2/ε3 variant	snRNA-seq 10x Chromium	Pan-astrocytic DEGs
Serrano-Pozo et al. 2022	Human	Entorhinal cortex, inferior temporal c., dorsolateral prefrontal c., visual association c., primary visual c.	AD	AD patients at multiple stages of the pathology	snRNA-seq 10x Chromium	astR1, astR2
Smajić et al. 2022	Human	Midbrain	PD	Idiopathic PD patients	snRNA-seq 10x Chromium	$CD44^{high}$
Al-Dalahmah et al. 2020	Human	Cingulate cortex	HD	Patients with grade III/IV HD	snRNA-seq 10x Chromium	Cluster 1, 2, 5, and 6
Wheeler et al. 2020	Mouse	Brain, spinal cord	EAE	Age ~2-4 months	scRNA-seq Drop-seq	MAFG
Sanmarco et al. 2021	Mouse	Spinal cord	EAE	Age ~2-4 months	scRNA-seq InDrop, Drop-seq	$LAMP1^{+}TRAIL^{+}$
Absinta et al. 2021	Human	Chronic active lesion edge in brain WM	MS	Patients with progressive MS	snRNA-seq 10x Chromium	AIMS, reactive/stressed astrocytes
Shi et al. 2021	Mouse	Brain regions affected by injury	Ischemic and hemorrhagic stroke	Age 10-12 weeks	scRNA-seq 10x Chromium	Cluster 3
Ma et al. 2022	Mouse	Cortex	Transient MCAO	Age 8 weeks, 12 h + 24 h post injury	scRNA-seq 10x Chromium	AST12_C, AST24_C
Zamboni et al. 2020	Mouse	Cortex	Stab wound injury	3-5 weeks post injury	scRNA-seq 10x Chromium	AC3
Li et al. 2022	Mouse	Spinal cord	SCI	Age 8 weeks, 0-38 days post injury	scRNA-seq 10x Chromium	Cluster 4 and 5
Wang et al. 2021	Rat	Spinal cord	SCI	1 day post injury	scRNA-seq Fluidigm C1	Cluster 4
Chancellor et al. 2021	Human	Dorsolateral frontal cortex	CTE	Patients with stage II or III CTE	snRNA-seq inDrops	Astrocyte2, Astrocyte3

Abbreviations: AD – Alzheimer's disease, AIMS – astrocytes inflamed in multiple sclerosis, CTE – chronic traumatic encephalopathy, DAAs – disease associated astrocytes, DEGs – differentially expressed genes, EAE – experimental autoimmune encephalomyelitis, hiPSC – human induced pluripotent stem cells, HD – Huntington's disease, iA – induced astrocytes, LPS – lipopolysaccharide, MCAO – middle cerebral artery occlusion, MS – multiple sclerosis, PD – Parkinson's disease, SCI – spinal cord injury, WM – white matter.
